# Validation of Sequential ROX-Index Score Beyond 12 Hours in Predicting Treatment Failure and Mortality in COVID-19 Patients Receiving Oxygen via High-Flow Nasal Cannula

**DOI:** 10.1155/2023/7474564

**Published:** 2023-02-08

**Authors:** Dimitris Basoulis, Pantelis Avramopoulos, Maria Aggelara, Georgios Karamanakos, Pantazis-Michail Voutsinas, Amalia Karapanou, Mina Psichogiou, Michalis Samarkos, Foteini Ntziora, Nikolaos V. Sipsas

**Affiliations:** ^1^Infectious Diseases and COVID-19 Unit, Athens, Greece; ^2^1st Internal Medicine Department, Athens, Greece; ^3^1st Propaedeutic Internal Medicine Department, Athens, Greece; ^4^Infection Control Nurse, Athens, Greece; ^5^Department of Pathophysiology, Medical School, National and Kapodistrian University of Athens “Laiko” General Hospital, Athens, Greece

## Abstract

**Background:**

High-flow nasal cannula (HFNC) is an oxygen delivery method shown to reduce the risk of intubation and mortality in patients with type 1 respiratory failure. The ROX-index score can predict HFNC failure. This study aims to evaluate sequential ROX-index assessments as predictors of HFNC failure and mortality.

**Methods:**

Prospective observational single-center study including all adult patients with positive SARS-CoV-2 PCR placed under HFNC from 1st November 2020 to 31st May 2021, and patients with hemodynamic instability or unable to tolerate HFNC were excluded. The primary endpoint was successful HFNC de-escalation.

**Results:**

In univariate analysis, HFNC de-escalation was associated with younger age (59.2 ± 14 vs. 67.7 ± 10.5 and *p* < 0.001), lower levels of serum lactate (1.1 vs. 1.5 and *p*=0.013), and higher ROX-index at 12 hrs (5.09 vs. 4.13 and *p* < 0.001). ROC curve analysis of ROX-index at 12 hrs yielded a c-statistic of 71.2% (95% CI 61.6–80.9 and *p* < 0.001). ROX-index at 12 hrs and age retained significance in multivariate analysis. Using an optimal cutoff point of 4.43, we calculated a sensitivity of 64.5% and specificity of 69.6%. In univariate survival analysis, older age (68.8 ± 9.7 vs. 58.9 ± 13.9 and *p* < 0.001), greater creatinine values (0.96 vs. 0.84 and *p*=0.022), greater SOFA score (*p*=0.039), and a lower 12 hrs ROX-index (4.22 vs. 4.95 and *p*=0.02) were associated with hospital mortality. The SOFA score and age retained significance in multivariate survival analysis.

**Conclusion:**

ROX-index is proven to be a valuable and easy-to-use tool for clinicians in the assessment of COVID-19 patients under HFNC.

## 1. Introduction

Until December 2019, treating patients with type 1 hypoxemic respiratory failure was a sporadic problem involving a limited range of specialties. The emergence of the novel coronavirus SARS-CoV-2 brought the whole medical society face-to-face with concepts, such as the decision for intubation and forced health professionals to quickly become accustomed to optimal oxygen therapy methods. The pressure of the Coronavirus disease 2019 (COVID-19) pandemic on even the most advanced health systems has led medical staff to increasingly use various oxygen therapy delivery systems and noninvasive ventilation (NIV) methods to provide optimal patient care amid a massive increase in the number of intubated patients [1].

The high-flow nasal cannula (HFNC) is a device that delivers high mixtures of oxygen (up to 100%), heated and humified at a maximum flow of 60–80 liters per minute, through a nasal cannula. Originally used in neonatal units, the HFNC is one of the most promising oxygen therapy methods used to treat adult patients with hypoxemia, due to its beneficial effects on the respiratory system, which have been extensively studied over the past decade [2]. In addition to reducing the work of breathing through generating low levels of positive airway pressure, reducing dead space and airway resistance, HFNC has been shown to be better tolerated by hypoxemic patients, with minimal and quickly reversible adverse effects, compared to face masks use [3]. Its ease of use and the capability of immediate improvement of PaO_2_ and respiratory rate upon its application, make it a favorable method of oxygen therapy, especially in low-resources environments as it reduces the risk of intubation, the total days under mechanical ventilation, and the total mortality of hypoxemic patients [4].

However, a main concern of HFNC application is the potential delay of intubation which is associated with increased mortality [5]. The ROX-index, which effectively refers to the ratio of oxygen saturation measured by pulse oximetry (SpO_2_)/fraction of inspired oxygen (FiO_2_) to the respiratory rate, is an accepted score that emerged during the recent years and has shown its value as a predictor of HFNC failure in patients with hypoxemia [6, 7]. A value greater than or equal to 4.88 at 12 hours after HFNC onset has a sensitivity of 70.1% and a specificity of 72.4% to predict HFNC failure. The main advantage of the ROX-index is its clinical score form without the need of lab results nor complex calculation methods. The ease of use fits the nature of hypoxemic COVID-19 patients care, as it gives the capability of quick and reliable assessment of multiple critically ill patients.

Nevertheless, the physicians' question, when to proceed to endotracheal intubation, remains. The aim of this study is to assess the ROX-index, along with other laboratory and clinical parameters, as prediction tools for the failure of HFNC in COVID-19 patients with hypoxemic respiratory failure to identify early those who may require invasive mechanical ventilation (IMV). We present the following article/case in accordance with the STROBE reporting checklist.

## 2. Materials and Methods

### 2.1. Study Design and Outcomes

This is an analysis of prospectively collected observational data from consecutive patients placed on HFNC (Airvo™2 by Fisher-Paykel) during hospitalization in the COVID-19 department of our hospital. Our primary endpoint was HFNC de-escalation, defined as HFNC withdrawal with improved oxygenation, no need for NIV and/or IMV. Secondary outcomes were the length of stay under HFNC treatment and overall, in-hospital mortality. Initial HFNC settings were for all patients 60 liters/min and 90% FiO_2_ with an SpO_2_ target of 92–96% and further titrated downwards based on needs after 12 hrs.

This study was approved by the Laiko General Hospital Scientific and Ethics Review Board (protocol number: 376/19-5-20). A written informed consent was obtained by all participants in the study.

### 2.2. Study Population

The total recruiting period was 7 months (from 1st November 2020 to 31st May 2021) encompassing Greece's 2nd and 3rd pandemic waves. The inclusion criteria were as follows: (a) age >18 years, (b) positive PCR test for SARS-COV-2 RNA, and (c) treatment with application of HFNC due to respiratory failure type I not responding to 10 L low-flow nasal cannula or up to 15 L and 60% FIO_2_ oxygen mask (our hospital did not have the option of noninvasive ventilation since the same devices used for it, were also used for mechanical ventilation and shortage of ventilators was a significant problem at the time). The exclusion criteria were as follows: (a) hemodynamic instability, (b) facial injuries preventing application of HFNC, (c) type II respiratory failure, and (d) patient inability to cooperate with HFNC.

### 2.3. Measurements

We recorded demographic data, relevant medical history, smoking habits, and laboratory and respiratory values on admission and on HFNC application and on several time-points thereafter (at 12 hours and on days 3, 5, 7, and 14). Sequential organ failure assessment (SOFA) [8] and ROX-index scores were calculated for these time-points. We also recorded information pertaining to treatment administered to these patients, prone positioning, HFNC treatment, and duration of symptoms and hospitalization. Bacterial pneumonia was defined as the presence of lobar pneumonia on x-rays and/or CTs as well as isolation of bacteria from sputum.

### 2.4. Statistical Analysis

Descriptive statistics are presented as counts (%) for categorical variables and as medians (25th–75th percentile) for non-normally distributed continuous variables or as means ± standard deviation (SD) for normally distributed continuous variables. The normality of distribution was examined using the Kolmogorov–Smirnov test. Group comparisons were performed using the student's *t*-test and Mann–Whitney or Wilcoxon signed-rank test for normally and non-normally distributed variables, respectively, chi-square for categorical variables and Spearman correlation for continuous variables' relationships. Multivariate analyses were performed using logistic regression to assess the probability of HFNC failure and Cox proportional hazards regression to assess hospital mortality. All variables with statistical significance, as defined by a *p* < 0.05 in the univariate analysis, were included. The results of the Cox model are presented as hazard ratios (HRs), while the results of the logistic regression as odds ratios (ORs), both with 95% confidence intervals (CIs) and with a statistical significance for *p* < 0.05. Finally, the validation of the ROX-index was performed using a received operating characteristic (ROC) curve analysis. The analysis was performed using SPSS Statistics for Windows, version 25.0 (2017, Armonk, NY, IBM Corp.).

## 3. Results

During the study period 1,115 patients were hospitalized in our COVID department for a total of 12,041 patient-days. Of these, 116 patients (10.4%) had critical type I respiratory failure and needed treatment with HFNC giving an incidence of 9.63 HFNC applications per 1000 patient-days. Five patients were unable to tolerate treatment with HFNC and were excluded from the statistical analysis. Our final analysis sample included 111 patients with mean age 62.8 ± 13.3 years. Most of our participants were male (70/111 and 63.1%) and 89.2% (99/111) of Greek descent. Baseline characteristics of our study population are presented in Table 1.

The primary outcome was met in 57.7% (64/111) of the participants. In univariate analysis, the only predictors of HFNC de-escalation were younger age (59.2 ± 14 vs. 67.7 ± 10.5 and *p* < 0.001), lower levels of serum lactate both on hospital admission (1.1 vs. 1.5 and *p*=0.013), and 12 hours after HFNC was applied (1.3 vs. 1.5 and *p*=0.007) and higher ROX-index scores on the 12-hour mark (5.09 vs. 4.13 and *p* < 0.001). A comparison between those who met the primary endpoint of HFNC de-escalation and those who did not is presented in Table 2. In multivariate analysis (Table 3), all three variables retained statistical significance.

ROX-index was calculated at 12 hours post-HFNC application and again at days 2 (*n* = 100), 3 (*n* = 84) and 7 (*n* = 35). We performed pair-wise comparisons (12 hr vs. d2, d2 vs. d3, d3 vs. d7, 12 hr vs. d3, and 12 hr vs. d7) of ROX-index scores using the Wilcoxon signed-rank tests. In the subgroup of patients that were not successfully de-escalated, there was no statistically significant difference in the median ROX-index scores at these timepoints (4.13 vs. 3.99 vs. 4.25 vs. 4.62). In the subgroup of patients that met the primary endpoint, however, there was significant improvement in scores when performing comparisons between the 12 hr-mark and day 7 (5.09 (4.05–5.87) vs. 5.67 (4.95–7.08) and *p*=0.016), between day 2 and day 7 (5.38 (4.26–6.39) vs. 5.67 (4.95–7.08) and *p* 0.017) and between day 3 and day 7 (5.16 (4.08–7.11) vs. 5.67 (4.95–7.08) and *p*=0.005). There was also improvement between the 12 hr-mark and day 2, which marginally did not meet statistical significance (5.09 vs. 5.38 and *p*=0.073). If we calculate the difference between ROX-index scores between these timepoints, we can derive a measure of ROX-index progression, which we can name DeltaROX. DeltaROX calculated between Days 7 and either day 2 or day 3 was found to have significantly different values between the subgroup of patients that was de-escalated and those that did not (0.68 vs −0.87 and *p*=0.016 and 0.92 vs −0.5, *p*=0.008, for day 7-day 2 and day 7-day 3, respectively). No other calculated deltas had statistical significance.

**Figure 1:**
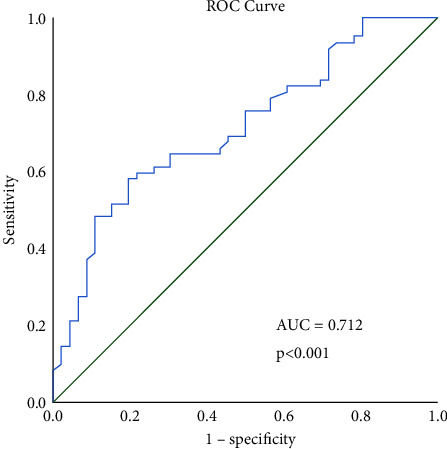
ROC curve for 12 hr ROX-index score predicting successful high-flow nasal cannula de-escalation.

The ROC curve analysis (Figure 1) for 12 hrs ROX-index to predict de-escalation yielded a c-statistic of 71.2% (95% CI 61.6–80.9 and *p* < 0.001). Using an optimal cutoff point of 4.43, we calculated a sensitivity of 64.5% and specificity of 69.6%. Optimizing for sensitivity, with a cutoff point of 3.85, sensitivity was 77.4% and specificity 43.5%, while optimizing for specificity, with a cutoff point of 4.88, sensitivity was 56.5% and specificity 80.4%.

Of the successfully de-escalated persons, four developed complications that later led to death, while seven patients, who had to be placed in IMV were eventually successfully discharged. In univariate survival analysis (Table 2), older age (68.8 ± 9.7 vs. 58.9 ± 13.9and *p* < 0.001), greater creatinine values on admission (0.96 vs. 0.84, *p*=0.022) and on the 12-hour mark (0.85 vs. 0.77, *p*=0.043), greater SOFA score on admission (*p*=0.039) and on the 12-hour mark (*p*=0.01), and a lower ROX-index score 12 hours after HFNC application (4.22 vs. 4.95, *p*=0.02) were associated with in-hospital mortality. In a multivariate Cox regression model, that included age, gender, SOFA, and ROX scores at the 12-hour mark, age, and SOFA alone retained statistical significance (Table 4). In the patient group that was intubated (*n* = 44) after HFNC failure, the duration of HFNC application prior to intubation did not differ significantly (*p*=0.6) amongst survivors (*n* = 37) and nonsurvivors (*n* = 7).

Patients that met the primary outcome had a median time under HFNC of 6 (4–8) days and median total duration of stay in-hospital of 15 (12–23)days. In contrast, patients that did not meet the primary endpoint stayed 3 (1–6) days under HFNC, a finding that met statistical significance (*p* < 0.001) compared to the primary endpoint group, while there was no difference in total duration of hospitalization with a median time of 16 (11–27) days. Amongst patients successfully de-escalated, there was a significant correlation between the ROX-index and the number of days spent under HFNC (Spearman *r* = −0.256 and *p*=0.044). Calculating the total days in-hospital introduces immortal time bias, so we compared the time from HFNC application till discharge or death and it did not differ significantly in the two populations (13 vs. 13 days and *p*=0.749).

## 4. Discussion

The current prospective study evaluated the ROX index and related factors as prediction tools for HFNC failure in COVID-19 patients with hypoxemic respiratory failure. We tried to include various factors to our analysis, some of which had not been examined as predictors for HNFC failure such as treatment with IL-6 receptor inhibition or complications such as pulmonary embolism and HAP. We also explored the ROX-index value as a daily assessment tool, through its calculation at 12 h and on days 2, 3, and 7 after HFNC initiation. Our findings indicate that the ROX-index is a valid predictor for HFNC failure and that the upturn of its values while under HFNC can serve as an indicator for successful de-escalation.

Throughout the COVID-19 pandemic, a major struggle for physicians treating COVID-19 patients with hypoxemic respiratory failure is the timing of endotracheal intubation and mechanical ventilation [9]. While the correlation of delaying intubation with higher mortality is evident from studies both preceding and following the COVID-19 outbreak [10, 11], shortage of ICU beds, ventilators and trained staff, even in developed countries, and presses physicians outside ICU to prolong patients' stay under HFNC/NIV treatment [9]. On the other hand, a recent meta-analysis comparing the outcomes of late and early intubations, showed no mortality benefit of either option [12]. Furthermore, endotracheal intubation is a procedure with multiple risks for COVID-19 patients and healthcare workers alike, while sedation and mechanical ventilation involve various complications [13]. The HFNC is proven to be a safe and effective way of oxygen supplementation for patients with severe hypoxemic respiratory failure [4]. Mellado-Artigas et al., demonstrated that HFNC might decrease ventilator days, ICU length of stay, and all-cause-hospital mortality of COVID-19 patients [14]. However, the implementation of HFNC as an alternative to mechanical ventilation could be a factor delaying escalation of care, resulting in unfavorable outcomes. Thus, the need for reliable and usable predictors of HFNC treatment failure is vital for aiding clinical judgement as well as managing available resources.

In this context, the ROX-index is turning to be one of the main focuses of researchers during the pandemic [6]. According to our results, the ROX value after 12 h in HFNC is a fair predictor of failure (AUC = 0.71) with a cutoff point of 4.43. It is also important to point out that a ROX-index value of 4.88 predicts HFNC failure with a specificity of 80.4%, possibly suggesting a more documented decision to follow a wait-and-see strategy, which is often arbitrarily implemented under the pressure of the pandemic, especially in resource-limited areas.

The day-to-day assessment of COVID-19 patients with severe hypoxemia under HFNC treatment is crucial to making the right therapeutic decisions. We suggest an alternative use of the ROX-index as a daily assessment tool, based on the significance of improvement of the ROX value between the first two days and the 7th day under HFNC among the success group and the absence of such improvement in the failure group. Early intubation should be considered when a patient's ROX value is not improving.

Serum lactate level increase is a well-known indicator of impaired tissue oxygenation [15]. The measurement of serum lactate is widely available, calculated directly from the ABGs analyzers that operate in many of the COVID-19 wards. Our study demonstrates that lactate is an independent factor of HFNC outcome, with higher levels suggesting a greater risk for intubation. Similarly, to our results, a small study from Baylor University in the US has also shown an association of lactate with HFNC failure [16].

Typical characteristics of the COVID-19 pneumonia is the sudden deterioration which can lead from mild to grave hypoxemia in a matter of hours, and the long time needed to recover once bilateral pneumonia has settled [17, 18]. In our study, there was no difference in total duration of hospitalization between the patients who were intubated and those who were successfully de-escalated from HFNC. Yet, the first group had a significantly shorter duration of stay under HFNC before intubation, in contrast with the second group who stayed longer under HFNC. This finding possibly reflects the sudden deterioration in the HFNC failure group and the absence of ROX-index improvement in the same patients. Amongst patients successfully de-escalated, there was a significant correlation between the ROX-index and the number of days spent under HFNC, further enhancing the utility of the ROX-index.

In addition to investigating predictors for HFNC treatment outcome, we also examined survival predictors for COVID-19 patients in HFNC. Our analysis included all the previously stated factors as well as the HFNC treatment duration and the SOFA score. Consistent with previous studies' findings, patients' age and SOFA score were shown to increase along with the mortality as independent risk factors [19, 20]. This finding indicates that using the SOFA score could help clinicians make rationalized and safe decisions during the management of hypoxemic COVID-19 patients outside the ICU.

A recent meta-analysis by Prakash et al. [21] shows that the validation of the ROX-index as a predictor of HFNC failure has already been established, yet most of the studies have been conducted in ICU environment and are retrospective. Suliman et al. [22] in a similar manner to our study, calculated the ROX-index over different days, as opposed to the first-few-hours approach of most researchers, but their study was not limited to patients under HFNC, including moderate cases of COVID-19.

Our study is not without limitations. Being a single-center study means that the number of patients enrolled cannot reach the high numbers of multicenter studies and involves the risk of poor demographic differentiation, therefore our findings might not be applicable to other settings. However, physicians who attend to COVID-19 patients could consider applying our findings, especially if the context of the study matches their own conditions. Secondly, some important prognostic factors for COVID-19 patients, such as lymphocyte count and D-dimers, were not recorded and thus not included in the analysis. The number of patients included in days 2, 3, and 7 is diminishing as patients have already met their primary endpoint at that time limiting the available data for these timepoints and any conclusions that could be reached. Finally, we did not calculate values of the ROX-index on days 4, 5, and 6, which could have resulted in more consistent findings regarding the prognostic value of the ROX-index progress during HFNC treatment.

## 5. Conclusions

The ROX-index is a valuable and easy to use tool in the everyday assessment of COVID-19 patients under HFNC. It could serve as an aide in deciding the optimal timing of intubation in patients with respiratory failure under HFNC and reduce mortality in this population. Larger randomized prospective studies are needed to investigate further its use.

## Figures and Tables

**Table 1:** Baseline characteristics and outcomes of study participants.

Variable	*N* = 111
Age, years, mean ± SD	62.8 ± 13.3
Male gender (%)	70 (63.1)
Greek descent (%)	99 (89.2)
COPD (%)	12 (10.8)
Type 2 diabetes (%)	28 (25.2)
Obesity (BMI>30) (%)	49 (44.1)
Active smoking (%)	14 (12.6)
Cardiovascular disease (%)	21 (18.9)
Chronic kidney disease (%)	4 (3.6)
Active malignancy (%)	3 (2.7)
Immunocompromised (%)	7 (6.3)
Hospital admission	
CRP, mg/l, median (IQR)	88 (48.1–141.3)
LDH, U/l, mean ± SD	444.97 ± 163.61
Lactate, mmol/l, median (IQR)	1.3 (0.9–1.7)
Ferritin, ng/ml, median (IQR)	751 (418–1106)
Platelets, x10^3^/*μ*l, median (IQR)	190 (150–245)
Creatinine, mg/dl, median (IQR)	0.84 (0.75–1.1)
Bilirubin, mg/dl, median (IQR)	0.49 (0.37–0.71)
SOFA median (IQR)	2 (2-3)
12 hrs after HFNC application	
CRP, mg/l, median (IQR)	75 (41.3–146.5)
LDH, U/l, mean ± SD	469.72 ± 182.69
Lactate, mmol/l, median (IQR)	1.3 (1–1.8)
Ferritin, ng/ml, median (IQR)	884 (545–1208)
Platelets, x10^3^/*μ*l, median (IQR)	233 (164–306)
Creatinine, mg/dl, median (IQR)	0.8 (0.68–1.01)
Bilirubin, mg/dl, median (IQR)	0.43 (0.33–0.77)
SOFA, median (IQR)	2 (2-3)
Respiratory rate, /min, median (IQR)	24 (20–28)
ROX-index and median (IQR)	4.43 (3.68–5.53)
Pulmonary embolism (%)	2 (1.8)
Bacterial pneumonia (%)	4 (3.6)
Treatment	
Prone position (%)	37 (33.3)
Tocilizumab (%)	48 (43.2)
Outcomes	
De-escalation (%)	64 (57.7)
Alive discharge (%)	67 (60.4)
Days under HFNC, median (IQR)	5 (2–8)
Days in hospital, median (IQR)	16 (12–26)

COPD = chronic obstructive pulmonary disease, BMI = body mass index, CRP = C-reactive protein, LDH = lactate dehydrogenase, SOFA = sequential organ failure assessment, and HFNC = high-flow nasal cannula.

**Table 2:** Demographic, clinical, and laboratory characteristics of patients according to the primary endpoint of HFNC de-escalation and according to hospital mortality.

Variable	Intubation/death (*n* = 47)	De-escalation (*n* = 64)	*p*	Death in hospital (*n* = 44)	Alive discharge (*n* = 67)	*p*			
Age, years, mean ± SD	67.7 ± 10.5	59.2 ± 14	<0.001	68.8 ± 9.7	58.9 ± 13.9	<0.001			
Male gender (%)	30 (63.8)	40 (62.5)	1	29 (65.9)	41 (61.2)	0.690			
Greek descent (%)	44 (93.6)	55 (85.9)	0.232	42 (95.5)	57 (85.1)	0.120			
COPD (%)	8 (17)	4 (6.3)	0.119	7 (15.9)	5 (7.5)	0.214			
Type 2 diabetes (%)	15 (31.9)	13 (20.3)	0.189	14 (31.8)	14 (20.9)	0.264			
Obesity (BMI>30) (%)	22 (46.8)	27 (42.2)	0.7	20 (45.5)	29 (43.3)	0.847			
Active smoking (%)	7 (14.9)	7 (10.9)	0.573	7 (15.9)	7 (10.4)	0.4			
Cardiovascular disease (%)	9 (19.1)	12 (18.8)	1	8 (18.2)	13 (19.4)	1			
Chronic kidney disease (%)	2 (4.3)	2 (3.1)	1	4 (9.1)	0	0.23			
Active malignancy (%)	2 (4.3)	1 (1.6)	0.573	2 (4.5)	1 (1.5)	0.561			
Immunocompromised (%)	3 (6.4)	4 (6.3)	1	5 (11.4)	2 (3)	0.111			
Hospital admission									
CRP, mg/l, median (IQR)	87 (51.88–150.3)	88.4 (42.1–138.5)	0.582	77.5 (52–158.2)	88.9 (41.7–140.8)	0.704			
LDH, U/l, mean ± SD	435.6 ± 144.3	451.9 ± 177.3	0.619	438.3 ± 161.3	449.3 ± 166.3	0.740			
Lactate, mmol/l, median (IQR)	1.5 (1.08–1.93)	1.1 (0.8–1.6)	0.013	1.3 (1–1.8)	1.25 (0.88–1.63)	0.467			
Ferritin, ng/ml, median (IQR)	780 (434–1154)	701 (411–1031)	0.410	825 (599–1182)	651 (357–1025)	0.114			
Platelets, x10^3^/*μ*l, median (IQR)	184 (144–238)	198 (151–247)	0.942	179 (140–235)	199 (152–247)	0.587			
Creatinine, mg/dl, median (IQR)	0.85 (0.75–1.11)	0.84 (0.74–1.09)	0.670	0.96 (0.79–1.15)	0.84 (0.73–1)	0.022			
Bilirubin, mg/dl, median (IQR)	0.49 (0.39–0.69)	0.48 (0.35–0.72)	0.823	0.47 (0.38–0.61)	0.53 (0.35–0.77)	0.423			
SOFA, median (IQR)	2 (2-3)	2 (2-3)	0.286	2 (2-3)	2 (2-3)	0.039			
12 hrs after HFNC application									
CRP, mg/l, median (IQR)	87 (51–199.7)	69.34 (35.8–124.1)	0.121	81 (53.5–209.7)	57.5 (32–133.8)	0.064			
LDH, U/l, mean ± SD	499.5 ± 152.8	448.6 ± 199.9	0.185	512.3 ± 168.2	442.1 ± 187.8	0.068			
Lactate, mmol/l, median (IQR)	1.5 (1.2–2.05)	1.3 (0.95–1.55)	0.007	1.5 (1.2–1.8)	1.3 (1–1.7)	0.249			
Ferritin, ng/ml, median (IQR)	845 (591–1201)	892 (511–1234)	0.906	971 (634–1316)	878 (464–1126)	0.264			
Platelets, x10^3^/*μ*l, median (IQR)	227 (152–287)	234 (165–325)	0.342	221 (157–274)	235 (164–340)	0.177			
Creatinine, mg/dl, median (IQR)	0.84 (0.62–1.1)	0.77 (0.68–1)	0.583	0.85 (0.71–1.42)	0.77 (0.67–0.93)	0.043			
Bilirubin, mg/dl, median (IQR)	0.54 (0.37–0.88)	0.4 (0.3–0.7)	0.114	0.52 (0.35–0.85)	0.43 (0.32–0.69)	0.423			
SOFA and median (IQR)	2 (2-3)	2 (2-2)	0.1	2 (2-3)	2 (2-2)	0.01			
Respiratory rate,/min, median (IQR)	25 (22–30)	22 (19–28)	0.012	24 (21–30]	24 (20–28)	0.269			
ROX-index, median (IQR)	4.13 (3.32–4.59)	5.09 (4.05–5.87)	<0.001	4.22 (3.62–5.05)	4.95 (3.78–5.83)	0.02			
Pulmonary embolism (%)	0	2 (3.1)	0.513	0	2 (3)	0.526			
Bacterial pneumonia (%)	2 (4.3)	2 (3.1)	1	1 (2.5)	3 (4.5)	1			
Treatment									
Prone position (%)	16 (34)	21 (32.8)	1	15 (34.1)	22 (32.8)	1			
Tocilizumab (%)	21 (44.7)	27 (42.2)	0.848	19 (43.2)	29 (43.3)	1			
Outcomes									
Alive discharge	7 (14.9)	60 (93.8)	<0.001						
HFNC de-escalation				4 (9.1)	60 (89.6)	<0.001			
Days under HFNC, median (IQR)	3 (1–6)	6 (4–8)	<0.001	4 (2–7)	6 (3–8)	0.016			
Days in hospital, median (IQR)	16 (11–27)	15 (12–23)	0.862	15 (10–25)	16 (13–27)	0.163			
Days from HFNC till discharge or death, median (IQR)	13 (9–25)	13 (10–21)	0.749	11 (7–21)	13 (10–25)	0.098			

COPD = chronic obstructive pulmonary disease, BMI = body mass index, CRP = C-reactive protein, LDH = lactate dehydrogenase, SOFA = sequential organ failure assessment, and HFNC = high-flow nasal cannula.

**Table 3:** Multivariate logistic regression analysis for prediction of successful HFNC de-escalation.

Variable	OR	95% CI	*p*
Age	0.94	0.9–0.98	0.005
Male gender	0.73	0.28–1.94	0.528
Lactate	0.4	0.17–0.93	0.032
ROX@12 hrs	1.97	1.29–3.03	0.002

HFNC = high-flow nasal cannula, OR = odds ratio, and CI = confidence interval.

**Table 4:** Multivariate Cox proportional hazards regression analysis for prediction of in-hospital mortality.

	HR	95% CI	*p*
Age	1.07	1.03–1.11	0.001
Male gender	1.08	0.53–2.23	0.826
SOFA	1.46	1.09–1.94	0.01
ROX@12 hrs	0.8	0.59–1.09	0.153

HFNC = high-flow nasal cannula, HR = hazards ratio, and CI = confidence interval.

## Data Availability

The data have been uploaded to the University of Athens repository, Pergamos, available at this link: https://pergamos.lib.uoa.gr/uoa/dl/object/3259142.
